# A Brief Review of the Role of 2D Mxene Nanosheets toward Solar Cells Efficiency Improvement

**DOI:** 10.3390/nano11102732

**Published:** 2021-10-15

**Authors:** T. F. Alhamada, M. A. Azmah Hanim, D. W. Jung, A. A. Nuraini, W. Z. Wan Hasan

**Affiliations:** 1 Northern Technical University, Mosul 41001, Iraq; thaerfaez@ntu.edu.iq; 2 Department of Mechanical and Manufacturing Engineering, Faculty of Engineering, Universiti Putra Malaysia, Serdang 43400, Selangor, Malaysia; nuraini@upm.edu.my; 3 Advanced Engineering Materials and Composites Research Center (AEMC), Faculty of Engineering, Universiti Putra Malaysia, Serdang 43400, Selangor, Malaysia; 4 Department of Mechanical Engineering, Jeju National University, 1 Ara 1-dong, Jeju 690-756, Korea; 5 Department of Electrical and Electronic Engineering, Faculty of Engineering, Universiti Putra Malaysia, Serdang 43400, Selangor, Malaysia; wanzuha@upm.edu.my

**Keywords:** power conversion efficiency, solar cells, MXenes, electrodes, additives, HTL/ETL

## Abstract

This article discusses the application of two-dimensional metal MXenes in solar cells (SCs), which has attracted a lot of interest due to their outstanding transparency, metallic electrical conductivity, and mechanical characteristics. In addition, some application examples of MXenes as an electrode, additive, and electron/hole transport layer in perovskite solar cells are described individually, with essential research issues highlighted. Firstly, it is imperative to comprehend the conversion efficiency of solar cells and the difficulties of effectively incorporating metal MXenes into the building blocks of solar cells to improve stability and operational performance. Based on the analysis of new articles, several ideas have been generated to advance the exploration of the potential of MXene in SCs. In addition, research into other relevant MXene suitable in perovskite solar cells (PSCs) is required to enhance the relevant work. Therefore, we identify new perspectives to achieve solar cell power conversion efficiency with an excellent quality–cost ratio.

## 1. Introduction

The development of innovative materials for efficient solar cells has garnered a lot of attention [1,2,3,4,5,6,7,8,9,10] because of the ever-increasing need for renewable and clean energy supplies [11,12,13,14,15]. Sunlight has been identified as the most prevalent, cheapest, and cleanest source of energy for meeting society’s long-term energy requirements. Solar cells convert sunlight directly into electricity—the most efficient and practical method to utilise solar energy. Earth-rich silicon (Si)-based solar cells dominate the industry, with power conversion efficiencies (PCEs) of over 26 percent and a 25-year average module living standard [16,17,18]. However, since Si solar cells have high initial production costs, researchers are turning their attention to less expensive alternatives, such as perovskite solar cells (PSCs), organic solar cells (OSCs), quantum dot solar cells (QDSCs), and dye-sensitised solar cells (DSSCs) [19].

PSCs are the most feasible option among these new PV technologies for providing a PCE equivalent to maturing silicon solar cells. Furthermore, compared to traditional Si-based technologies, their lower costs, adjustable band gap, processability at low temperatures, long charge carrier diffusion lengths, high light absorption coefficients, lower exciton binding energy, numerous options for much simpler mass production processes lacking additional advantages, and increasing performance make it a more lucrative option [20,21,22,23,24,25,26]. Additionally, in contrast to traditional Si solar cells, PSCs operate well even in diffuse or weak light, making them suitable for specialised purposes [27]. Due to the development of various architectures, chemical compositions, manufacturing protocols, advances in materials, and phase stabilisation techniques, efficiencies have increased dramatically since the first report on all-solid-state PSCs in 2012, from 9.7% in 2012 to 25.5% percent in 2021 [28,29,30,31]. Between the highest observed efficiency and its theoretical maximum, PSCs may be split into two categories: the normal (n-i-p) structure and the inverted (p-i-n) structure [32,33].

Furthermore, concerns regarding PSC stability have been highlighted because a thin-film solar device must pass the IEC 61,646 environment stability test before it can be sold commercially [34]. A lot of research is now underway to improve the stability and performance of PSCs [19]. Scientists have been attempting to integrate perovskite into solar cells since the material’s initial breakthrough in 2009. The solar cells in this material are more efficient than those in current solar modules [35]. On average, existing solar modules capture 15 to 18 percent of the sun’s energy, while perovskite solar cells have an efficiency of up to 28 percent [36]. Dou’s research team developed a sandwich-like material that mixes organic and inorganic components to form a composite structure that does not need lead and improves stability considerably. According to Yao Gao, the new organic–inorganic hybrid perovskite materials are cheaper and perform better than traditional inorganic semiconductors. Solar cells can be highly efficient using this new method; the authors made hybrid perovskite materials that are intrinsically more stable. These novel materials are better for the environment and safer for bioelectronic sensors on humans because the researchers removed hazardous lead [37].

Transition-metal nitrides, or carbides (MXenes), were first found in 2011 by Gogotsi and his coworkers as star materials from MAX phases, which are layered compounds resembling graphite with monoatomic A element layers sandwiched between electrically conductive and stiff MX-blocks [38,39,40]. It was proposed that the generated material be labelled MXenes to highlight the removal of the A element from the MAX phase and its two-dimensional (2D) shape, related to graphene. The material has recently shown promising applications in solar cells [41,42,43,44], biomedical fields [45,46,47], light-emitting diodes [48,49,50], sensors [51,52,53,54,55], energy storage [56,57,58,59,60,61,62], catalysis [63,64,65,66], water purification [67,68,69,70,71,72], and electromagnetic applications [41,42,43,44,73]. The nanoengineering of these 2D materials is a hot topic right now. Due to its adjustable work function, high electrical conductivity, good transparency, and charge-carrier mobility, Ti_3_C_2_T_x_ (T stands for certain surface-terminating functional groups such as O, OH, and F) leads the current research on MXene in solar cells [74,75,76]. MXenes are currently divided into transition metals in either an out-plane or in-plane ordered form. Furthermore, most 2D transition-metal MXenes exist in the form of random solid solutions, which are characterised by two randomly distributed transition metals across the 2D structure. This review paper detailed the basic principles for the creation of each 2D transition-metal MXene structure, as well as their tunable characteristics depending on the transition-metal composition. 2D transition-metal MXenes vary from their counterparts mono-transition-metal MXenes, where two transition metals can occupy the metal sites.

**Figure 1:**
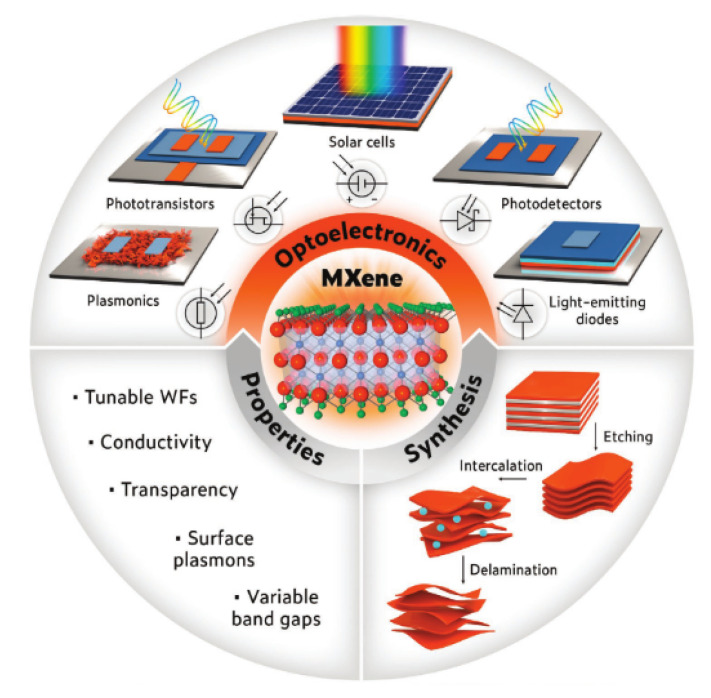
MXene synthesis, properties, and their applications. Reprinted with permission from ref. [73]. Copyright 2021 John Wiley & Sons, Inc.

Guo and his group included Ti_3_C_2_T_x_ as an additive in the photoactive layer of methylammonium lead iodide (MAPbI_3_) in the first research on MXene materials in perovskite solar cells, which was published in 2018 [77]. Since then, its application has been extended to the electrode, electron transport layer (ETL)/hole transport layer (HTL). The Ti_3_C_2_T_x_ functions on MXenes in solar cell applications may be classified into three categories: electrode [78], additive [77], and ETL/HTL [79,80]. Figure 1 below summarises the synthesis, properties, and application of MXene. The solar cells (SCs) in Figure 1 have been widely investigated [73].

This article summarises all previously reported work on incorporating MXene into solar cells to improve solar power generation and operational stability. The next section defines the efficiency improvement of SC and how it is classified. Section 3 lists the types of roles that MXene mainly plays in solar cells. A conclusion and prospect are given in Section 4.

## 2. The Efficiency Improvement of Solar Cells

The conversion efficiency of a solar cell is a measurement of incident light that can be converted to electrical energy. The incident light’s power is the denominator, while the solar cell’s electrical power is the numerator; thus, this conversion efficiency may be expressed as a fraction [77].

The power conversion efficiency (PCE) of solar cells is one of the most significant parameters [81]. The PCE has improved rapidly since the PSC’s introduction in 2009 [82]. The PCE of Kojima et al. initial’s PSC was just 3.8 percent [83]. Im et al. [84] claimed a PCE of 6.5 percent in 2011, while Kim et al. [28] recorded a PCE of nearly 9 percent in 2012. In 2016, approximately 22% of PCEs were verified, to the best of our knowledge [85]. All of these remarkable outcomes and conclusions in such a short period of time demonstrated PSC’s tremendous potential [86]. Below is the equation used to calculate the conversion efficiency:

Conversion efficiency (%) = Generated electrical power (W)/Incident light power (W) × 100(1)

Fu et al. published a paper in 2019 that can be applied to various MXene compositions as possible electrodes for the creation of high-performance solar cells. Solar cells with a maximum power conversion efficiency (PCE) of 11.5 percent were delaminated from a few stacked Ti_3_C_2_T_x_ MXene-contacted Si layers [87]. The authors recently suggested integrating an inorganic 2D Cl-terminated Ti_3_C_2_ (Ti_3_C_2_Cl_x_) MXene into the volume and surface area of CsPbBr_3_ lm to substantially decrease the superficial lattice tension. The faulty surface is healed, and a champion efficiency of 11.08 percent is obtained with an ultra-high open-circuit voltage of up to 1.702 V on the fully inorganic CsPbBr_3_-PSC, which is the greatest efficiency record for this kind of PSC to date. In addition, at 80 percent relative humidity for 100 days and 85 degrees Celsius for 1 month, the unencapsulated device performs nearly as well as the enclosed device [86]. Y. Zhang et al. used density functional theory calculations to thoroughly assess 64 2D transition-metal carbide (MXene) to determine that they were acceptable semiconductors for solar cells via material screening. Ti_2_CO_2_/Zr_2_CO_2_ and Ti_2_CO_2_/Hf_2_CO_2_ heterostructure solar cells, in particular, have really high power conversion efficiency of 22.74 and 19.56 percent, respectively (Most PCEs inside this paper were evaluated at AM-1.5G-illumination). This research opens the path for MXenes to be used as solar materials in the future [88]. According to Saeed et al., many new opportunities for creating effective indoor organic photovoltaics (OPVs) for practical applications can be explored. With the introduction of different optoelectronic methods to improve device performance under low indoor lighting with varied spectra, the indoor efficiency of OPVs (for PCE > 30%) has taken a quantum leap [89]. Saeed et al. demonstrated additional enhancements to dye-sensitised photovoltaic cells (DSPVs) in indoor solar applications for light energy recycling due to its outstanding light-harvesting performance under ambient lighting conditions. DSPVs’ suitability for ambient energy harvesting is proven by their record high power conversion efficiency (PCE) of over 30% under indoor lighting circumstances, consistent device operation, cost-effectiveness, colorful aesthetics, and PCE retention of up to 99% [90].

## 3. Applications of MXene in Solar Cells

### 3.1. MXene as Conducting Additives in Solar Cells’ Photoactive Active Layer

MXene as a photoactive layer additive in SCs is discussed in this section. Despite significant advances in PCE, charge-carrier recombination inside of the photoactive layer and at perovskite/ETL and perovskite/HTL interfaces still limits PSC performance. Improvements in charge-carrier management are essential to closing the gap between the existing PCEs and the theoretic efficiency frontier of CSs. Prior to mass manufacturing, the intrinsic instability of perovskite in humidity and at high temperatures, as well as the device’s limited scalability, must be addressed. Two-dimensional nanomaterials with distinct characteristics have been investigated as additions in photoactive perovskite layers of the HTL/ETL of PSC in recent years. The use of additive engineering to enhance the surface coverage and crystallisation of perovskite films has proven to be successful.

Guo et al. investigated the inclusion of Ti_3_C_2_T_x_ in the MAPbI_3_-based perovskite absorber for the first time in 2018 [77], kicking off research on MXenes in solar cells. Their findings indicate that adding Ti_3_C_2_T_x_ to MAPbI_3_ may prolong the nucleation process, resulting in larger crystals. Furthermore, the Ti_3_C_2_T_x_ additive is extremely helpful in speeding electron transport across the grain boundary, similar to a carrier bridge [91,92,93,94]. This is measured by the reduced charge-transfer resistance for the Ti_3_C_2_Tx additive, as revealed by the electrochemical impedance spectra. The median power conversion efficiency (PCE) rises from 15.2 percent to 16.8 percent because of these factors. In addition to adding Ti_3_C_2_T_x_ to the photoactive MAPbI_3_ layer, similarly, Agresti et al. added Ti_3_C_2_T_x_ to the TiO_2_/ETL to fine-tune its work function (WF). This lowered it from 3.91 to 3.85 eV, which is beneficial for tuning the interfacial energy levels between the perovskite absorber and the TiO_2_/ETL, improving charge transfer and lowering the barrier height. The device achieves a PCE of 20.14 percent, which is 26.5 percent greater than the control device without the Ti_3_C_2_T_x_ addition, thanks to the double addition and optimisation of both the photoactive MAPbI_3_ and the TiO_2_ electron transport layer. Furthermore, the inclusion of Ti_3_C_2_T_x_ to the current density-voltage (JV) curves was shown to decrease hysteresis while enhancing the PSCs’ long-term exposure stability. Recently, this group used density functional calculations to further investigate the MAPbI_3_ perovskite/Ti_3_C_2_T_x_-based MXene interface. When the relative concentrations of the OH, O, and F termination groups were changed, the findings indicate that the work function interface displays highly nonlinear behaviour, and they offer a profound insight into the alignment of the energy level for the manufacture of high-performance materials [15].

Agresti et al. used Ti_3_C_2_T_x_ MXene in MAPbI_3_ PSCs to modify the work function of perovskite films and ETLs, resulting in a power conversion efficiency improvement of 26%, as compared to Ti_3_C_2_T_x_-free control devices [95]. Di Vito and his colleagues used DFT to conduct first-principles calculations on a Ti_3_C_2_/MAPbI_3_ perovskite-coupled system, linking WF tuning to changes in the various concentrations of OH-, O-, and F-MXene- Terminations, and found that OH collections had the greatest impact in reducing work function [94].

Zhang and his colleagues used an in situ solution growth technique to synthesise MAPbBr_3_ nanocrystals (NCs) on the surface of multilayer MXene (Ti_3_C_2_T_x_) nanosheets that form heterostructures in 2020 [96]. PSCs were manufactured utilising the C-TiO_2_/m-TiO_2_-TQD/TQD-Perovskite/Spiro-OMeTAD-Cu_1.8_S design to enhance PCE and device stability while retaining a champion hysteresis-free power conversion efficiency of 21.64% compared to 18.31% for control devices, with substantially better long-term air and light stability. The entire potential of MXene materials in SCs must be explored as a new area. Various groups, on the other hand, revealed different methods for making use of 2D MXene materials’ higher electrical conductivity. 2D Ti_3_C_2_T_x_ MXene nanosheets were used as nanoscale additives in 2D Ruddles-den-Popper PSCs by Jin et al. The PCE of 2D PSCs rose from 13.69 percent (control device without MXene additive) to 15.71 percent [97] due to passivated trap states, optimal orientation, reduced charge transfer resistance, and enhanced crystallinity. Yang et al. utilised SnO_2_-Ti_3_C_2_ MXene nanocomposites as electron transport layers (ETLs) in planar PSCs [98].

**Figure 2:**
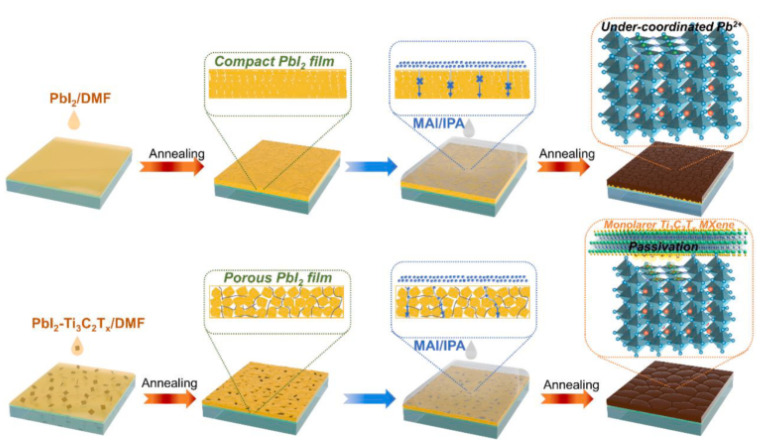
Mechanism diagram for the production of high-quality perovskite films processed in two steps, supported by the additive Ti_3_C_2_T_x_. Reprinted with permission from ref. [100]. Copyright 2020 Elsevier B.V.

Zhao et al. utilised Ti_3_C_2_T_x_ MXene nanosheets as a multifunctional additive in a two-step method to create extremely efficient planar PSCs in 2021. The findings indicate that single-layer Ti_3_C_2_T_x_ nanosheets improve the reactivity of the PbI_2_-layer by inducing the formation of a porous PbI_2_-layer, which increases the perovskite grain size and lowers the amount of residual PbI_2_ in the perovskite film. Random stacking of large PbI_2_ grains readily leads to the formation of pores, according to previous research [99]. The mechanism diagram to produce high-quality perovskite films is shown in Figure 2. Ti_3_C_2_T_x_ can also improve the WF of MAPbI_3_, allowing for better energy-level alignment between the perovskite layer and the ETL. Finally, by interacting with the under-coordinated Pb_2_+, the terminal collections on the surface of Ti_3_C_2_T_x_ play a critical role in the passivation of perovskite films. The maximum PCE of 16.45 percent and a PCE rate of 15.94 percent were obtained at the optimum Ti_3_C_2_T_x_ dose of 0.03 percent by weight. These values are about 18 percent better than those of pure PSCs, which had the greatest power conversion efficiency of 16.45 percent and a PCE rate of 15.94 percent. As a result, this research established Ti_3_C_2_T_x_ as an effective and feasible addition for the manufacture of greatly efficient two-stage produced PSCs, paving the path for their application to other 2D materials [100].

Larciprete et al. investigated pure environmental aging and the thermally driven breakdown of the mixed halide perovskite Csx (FA_0.83_MA_0.17_) (1x) Pb_3_ using X-ray photoelectron spectroscopy (I_0.83_Br_0.17_) and high-resolution ultraviolet. The scientists also looked at the impacts of the Ti_3_C_2_T_x_ MXene additive on photovoltaic stability as part of their research. Furthermore, the absence of any negative impact on PV stability, as well as a significant stabilising effect of the additional MXene, contribute to long-term aging. In the fresh samples, we observed a modest decrease in the initial halide migration rate, but this needs more investigation. In conclusion, we believe that our findings on Csx (FA_0.83_MA_0.17_) (1x) Pb_3_ (I_0.83_Br_0.17_) show severe criticality in the stability of certain mixed perovskites that are comparable to single-halide materials. As a result, it appears that the effectiveness of agents based on electronic and chemical stabilisation of their functional properties, as well as the creative development of device architectures capable of interacting with disruptive agents, are critical for the long-term use of mixed perovskite [101].

**Figure 3:**
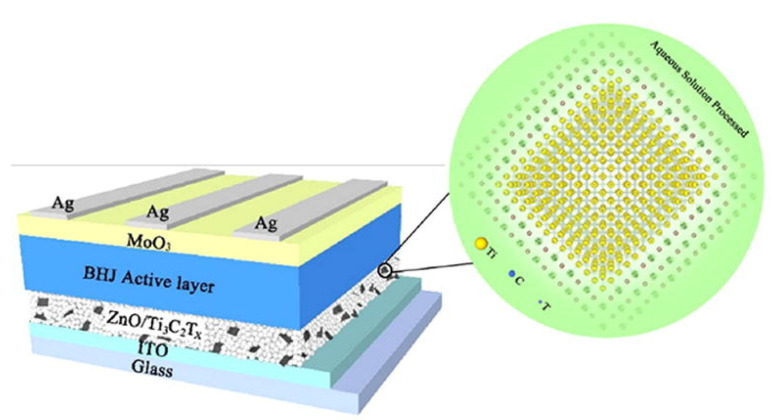
Schematic representation of the IPSCs configuration. Reprinted with permission from ref. [102]. Copyright 2020 Elsevier B.V.

**Figure 4:**
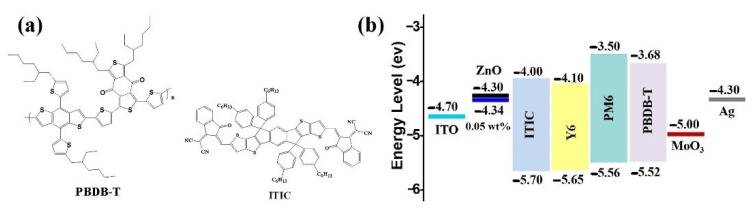
(**a**) Chemical structures of PBDB-T and ITIC. (**b**) Band diagram of the materials used in IPSCs. Reprinted with permission from ref. [102]. Copyright 2020 Elsevier B.V.

For the first time, Hou & Yu showed further improved IPSCs using Ti_3_C_2_T_x_ nanosheets as an additive in ZnO. The creation of the Zn–O–Ti bond enhances the PCE when ZnO is modified with Ti_3_C_2_T_x_, because of the recently created charge transfer routes between both the passivated surface of ZnO films and the ZnO nanocrystals. Figure 3 and Figure 4 illustrate energy level diagrams of the materials utilised in IPSCs. When compared to the control device that utilises pure ZnO as ETL, ITIC-based IPSCs with ZnO/Ti_3_C_2_T_x_/ETL achieve an average power conversion efficiency of 12.20 percent, which is a 15.53 percent improvement (10.56 percent). PM6: Y6 IPSCs reach a champion power conversion efficiency of 16.51 percent based on the ZnO/Ti_3_C_2_T_x_ interface layer, compared to 14.99 percent for the reference device [102].

**Figure 5:**
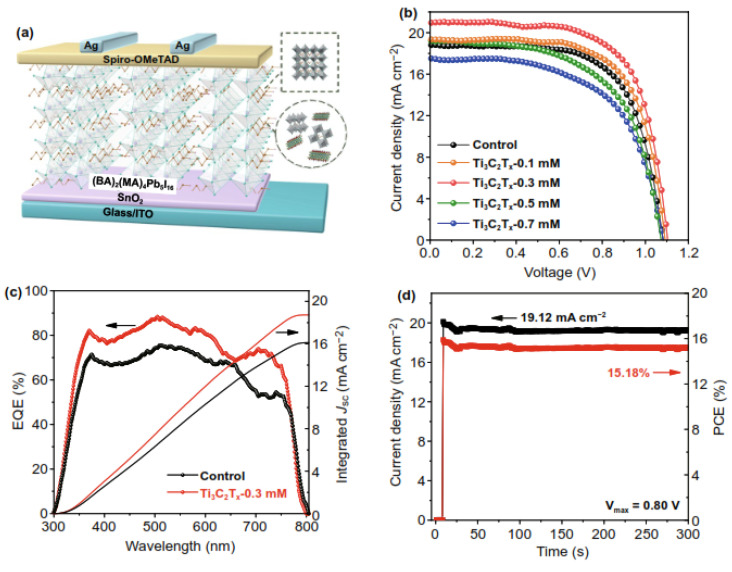
(**a**) Schematic representation of devices with the structure glass/ITO/SnO_2_/2D perovskite/SpiroOMeTAD/Ag. (**b**) JV curves from devices with different amounts of Ti3C2Tx doping. (**c**) EQE spectra and integrated Jsc of the control and optimised Ti_3_C_2_T_x_ doping devices. (**d**) Stabilised power output and current density at a constant bias 0.80 V for the Ti_3_C_2_T_x_ dopant devices. Reprinted with permission from ref. [99]. Copyright 2021 Springer Nature Switzerland AG. Part of Springer Nature.

According to Jin et al., a modest doping level of Ti_3_C_2_Tx nanosheets significantly enhanced the quality of 2D perovskite (BA) 2 (MA) 4Pb_5_I_16_ films and the photovoltaic performance of the associated device, with a PCE increase from 13.7 to 15.7 percent due to the increase in current. Figure 5a depicts the architecture of the current PSCs, as well as an example of Ti_3_C_2_T_x_ incorporation into a 2D perovskite film. Figure 5b shows the JV curves of the devices constructed using the control, Ti_3_C_2_T_x_0.1 mM, Ti_3_C_2_T_x_0.3 mM, Ti_3_C_2_T_x_0.5 mM, and Ti_3_C_2_T_x_0.7 mM samples. The external quantum efficiency (EQE) spectrum displayed in Figure 5c supports this growth in short-circuit current density (Jsc). Furthermore, a steady power output compatible with the JV curves is shown by the photocurrent evaluated for much more than 5 min at a point of maximum power (0.80 V) (Figure 5d). The enhanced vertically directed growth, uniform phase distribution in the thin film, and the crystallinity, which eventually improves charge transfer, are primarily responsible for the Ti_3_C_2_T_x_-doped components’ superiority. Furthermore, owing to the superior crystallinity and passivation effect of the perovskite film, the components doped with Ti_3_C_2_T_x_ nanosheets had a greater moisture stability than the shell components [99]. We can conclude that MXene has many functions in solar cells. As an additive, it accelerates electron transport by acting as an “electron” bridge. Hence, by its addition, it influences the carrier transport materials’ work function and other characteristics like conductivity. This research offers a viable approach for enhancing the efficiency of 2D perovskite film and expands the scope of Ti_3_C_2_T_x_’s photovoltaic applications [99].

### 3.2. Novel Metal Transparent Conductive Electrode

In PSCs, MXene is used as an electrode. An electrode is one of the most essential components of a PSC for controlling the charge collecting process; it is important for long-term stability and affects the device’s overall cost. Metal thin-film electrodes, nanostructured metal electrodes [103], carbon electrodes [104], and graphene electrodes [105], Ref. [106] are some of the newly described electrode materials for PSC.

The Ti_3_C_2_T_x_ MXene recently reported an electrical conductivity of up to 15,100 S cm^−1^ [107], as well as great transparency, good flexibility, and tunable WF [108,109,110]. Because of these characteristics, Ti_3_C_2_T_x_ may be used as an electrode in optoelectronic devices such as solar cells. The next sections elaborate on Si-wafer-based, organic, perovskite-based, and dye-sensitised solar cells, in that sequence. In quantum-dot-sensitised solar cells, the Ti_3_C_2_T_x_ MXene was also utilised to make the counter electrode (CE) (QDSCs). Chen et al. described a hybrid CE made up of hydrothermally produced CuSe nanoparticles on Ti_3_C_2_T_x_-MXen nanosheets screen printed on graphite foil [111]. This composite CE offers higher electrical conductivity for electron transport and a greater specific surface area than CuSe and Ti_3_C_2_T_x_-based CEs, allowing for more active centers for polysulfide electrolyte reduction. The device can obtain a PCE of 5.12 percent by employing a CuSe- Ti_3_C_2_T_x_ hybrid CE with an optimum mass ratio. Devices that utilise CuSe and Ti_3_C_2_T_x_-based CEs, on the other hand, have a PCE of 3.47 percent and 2.04 percent, respectively. Similarly, Tian et al. used a simple ion-exchange technique at ambient temperature to produce CuS/Ti_3_C_2_ composite CEs, which exhibited a substantially higher electrocatalytic rate for polysulfide reduction than pure CuS [112]. The overall PCE of the QDSC based on this composite CE is 5.11 percent, which is 1.5 times higher than that of a device with pure CuS CE. The combined benefits of the Ti_3_C_2_ framework’s high conductivity and the numerous catalytically active centers of the CuS nanoparticles are mostly responsible for the improved performance [15].

Cao et al. utilised 2D MXene material (Ti_3_C_2_) as a back electrode in non-precious metal PSCs and hole-transport materials in 2019 [78]. This increase in PCE was ascribed to the Ti_3_C_2_ electrode’s superior charge extraction capacity and reduced square resistance when compared to carbon electrodes. Jiang and his colleagues recently reported that, by using a combination of one-dimensional carbon nanotubes (CNTs), two-dimensional Ti_3_C_2_-MXene nanosheets, and commercial carbon paste as the electrode material in CsP-bBr_3_-PSC, they were able to obtain a power conversion efficiency of 7.1% [19,113].

In dye-sensitised solar cells, the 2D-layered Ti_3_C_2_ counter electrode substantially surpassed V2C in 2021 when compared to the iodide redox couple. According to Xu et al., the catalytic activity of Ti_3_C_2_ may be enhanced by increasing the etching time suitably. A PCE of 6.2 percent was found in DSCs with a Ti_3_C_2_ counter electrode etched for 24 h. Furthermore, K + intercalation has the potential to substantially boost Ti_3_C_2_’s catalytic activity, which is affected by the increased number of catalytic activity centers and the increased interlayer spacing for smooth iodide electrolyte transport. The PCE of the DSCs with the K + -Ti_3_C_2_ counter electrode was 7.11 percent, which was notably similar to the PCE of the conventional DSCs using Pt counter electrodes (7.2%) [114]. Chen et al. made the first effort to utilise MXene/CoS as an electrocatalytic CE for QDSSCs in their research. When compared to QDSSCs with bare MXene (4.25%) and bare CoS (5.77%) CEs, the QDSSCs with an Mxene/CoS/CE exhibit a substantial improvement in cell performance and provide a promising PCE of 8.1% [115].

**Figure 6:**
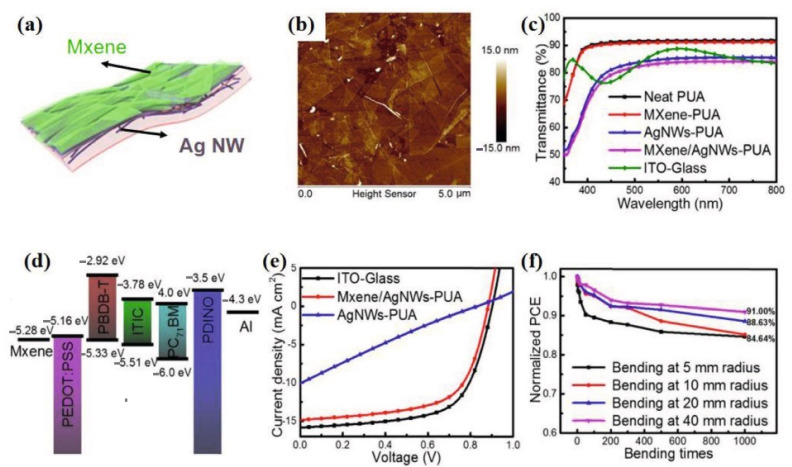
(**a**) Schematic representation of the MXene/AgNW hybrid electrodes on PUA substrates. (**b**) AFM images of the MXene/AgNW PUA films. (**c**) Transmission spectra of pure PUA, MXene-PUA, Ag NW-PUA, optimised MXene/Ag NW-PUA, and ITO glass. (**d**) Energy level diagrams of the flexible OSCs. (**e**) JV curves of the flexible OSCs with PBDB-T: ITIC: PC71BM active layers. (**f**) Normalised PCE of the flexible OSCs with MXene/Ag NW electrodes as a function of the number of bending cycles. Reproduced with permission. Reprinted with permission from ref. [116,117]. Copyright 2019 American Chemical Society.

Additionally, a fan was installed to aid in the construction of flexible OSCs. This study emphasises the significance of developing FTEs and demonstrates their essential importance in flexible OSCs. With a sheet resistance of 110 sq^−1^, the transparent Ti_3_C_2_T_x_ Mxene electrodes have the lowest sheet resistance to date. As a result, scientists and engineers should collaborate to develop FTEs with the high electrical and optical compromise needed for highly efficient flexible OSCs. Tang et al. [116] demonstrated a flexible non-fullerene OSC with Ag NW/Mxene component electrodes and PBDB-T: ITIC: PC71BM active layers utilising the Ag NW/Mxene component electrodes (Figure 6) [117].

**Figure 7:**
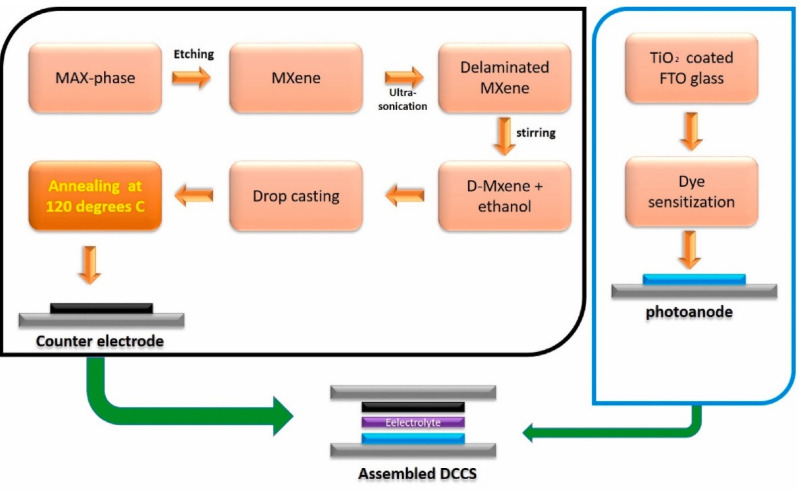
Schematic representation of an experimental procedure. Reprinted with permission from ref. [118]. Copyright 2021 Elsevier Ltd. and Techna Group S.r.l.

Ahmed et al. studied the application of single-layer delaminated 2-D-MXene (Ti_3_C_2_) created by the leaching method to replace both TCO and Pt as a conductive layer and a catalyst. Each test required at least five samples. To prevent human error and obtain the greatest possible conversion efficiency for reliable comparisons, a pre-built TCO Pt meter was utilised as the reference counter electrode (CE). Figure 7 depicts the whole procedure. Furthermore, Ti3C2 was adjusted in thickness for optimum conversion efficiency. At optimum thickness, the TCO/Pt/free MXen-based CE had a PCE of 8.68%, which was 4.03% higher than the conventional TCO/Pt-based counter electrode. The high efficiency is attributable to the high conductivity, the large number of accessible catalytic centers owing to the delaminated structure, and Ti_3_C_2_’s excellent catalytic activity towards iodide and triiodide electrolytes [118].

Hence, we can conclude that MXene serves a variety of roles in solar cells. As an electrode, it improves the form of hybrid electrodes with other conducting nanomaterials, such as metallic nanowires or carbon nanotubes. In addition, it enhances transparency, increases flexibility, metallic conductivity, and influences the work functions.

### 3.3. Mxene as Transfer Layer HTL/ETL in Solar Cells

**Figure 8:**
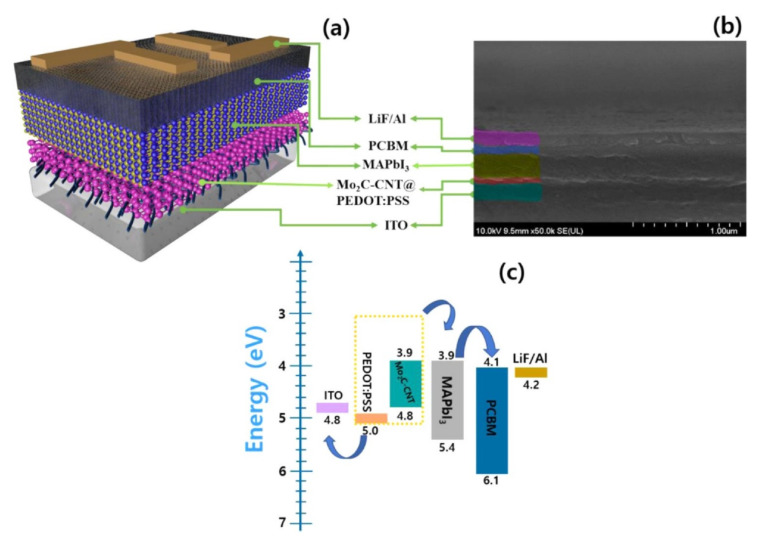
(**a**) Device architecture of the prepared ITO/HTL/CH_3_NH_3_PbI_3_/PCBM/LiF/Al prototype using Mo_2_C-CNTs @ PEDOT: PSS HTL and (**b**) FESEM cross-sectional image; (**c**) Energy level diagram for ITO/Mo_2_C-CNTs @ PEDOT: PSS/CH_3_NH_3_PbI_3_/PCBM/LiF/Al structure. Reprinted with permission from ref. [121]. Copyright 2021 Elsevier B.V.

The Electron Transport Layer (ETL) and Hole Transport Layer (HTL) in perovskite solar cells play an essential role in increasing stability (PSCs) and photovoltaic performance. The ETL’s primary function is to collect and transmit electrons from the perovskite layer while also preventing hole backflow, efficiently segregating charges, and reducing charge recombination [119]. The HTL’s primary function is to collect and transport holes from the photoactive perovskite layer to the electrode while also acting as an energy barrier to inhibit electron transmission to the anode. Furthermore, the HTL efficiently divides the photoactive perovskite layer from the anode and isolates air moisture, which enhances the stability of PSCs by reducing deterioration and corrosion [120]. The HTL PSC performance of component prototypes with various Mo_2_C @ CNT nanocomposite loading (1, 1.5, and 2 wt.-percent) was also investigated. Then, the Mo_2_C-CNT @ PEDOT: PSS HTL-based device was utilised as an X-ray photodetector, with a maximum sensitivity of 3.56 mA/Gycm2. Figure 8a depicts the schematic structure of the ITO/HTL/CH_3_NH_3_PbI_3_/ETL/LiF/Al-PSC using Mo_2_C-CNT @ PEDOT: PSS as HTL in the ITO/HTL/CH_3_NH_3_PbI_3_/ETL/LiF/Al-PSC using Mo_2_C-CNT @ PEDOT: PSS as HTL. The architecture of this composite perovskite solar cell was studied using cross-sectional FESEM (Figure 8b), and the associated energy level diagram is presented in Figure 8c. The findings show that Mxene/CNT nanocomposites with a perovskite layer have the potential to improve the efficiency of SCs and photodetectors. A high PCE of 11.98 percent was obtained for the HTL containing 1.5 percent by weight Mo_2_C-CNTs mixed with PEDOT: PSS in a component architecture of ITO/HTL/CH_3_NH_3_PbI_3_/PCBM/LiF/Al, which is greater than the HTLs with Mo_2_C (9.82%) and CNT (10.61%) mix [121].

**Figure 9:**
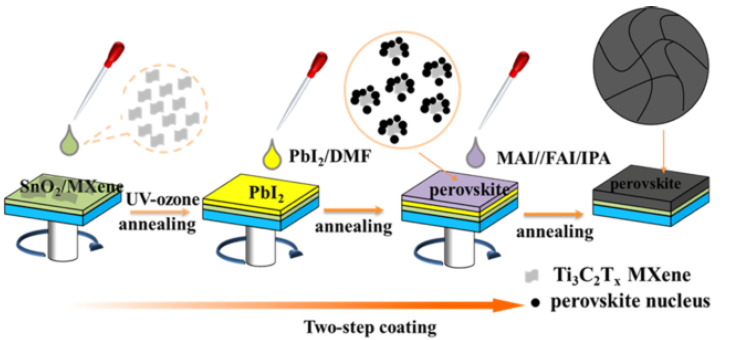
Schematic representation of the manufacturing processes of the perovskite film and the ETL. Reprinted with permission from ref. [123]. Copyright 2021American Chemical Society.

According to Bati et al., the incorporation of 2D MXenes into the ETL of PSCs produces extremely effective photovoltaic (PV) components. A power conversion efficiency of over 21% is obtained with the optimum composition [122]. In a planar PSC with a regular structure, Zheng et al. examined a hybrid film of SnO_2_ nanoparticles and Ti_3_C_2_T_x_ MXene nanoflakes as an electron transport layer (ETL). The ETL and perovskite layer production procedures are shown in Figure 9. The results show that the film qualities of the upper perovskite layers can be controlled by changing the Ti_3_C_2_T_x_/SnO_2_ ratios (2.02 wt percent in ETLs), such as crystallinity, crystal size, compactness, defect density, optical absorption, surface roughness, and so on, by changing the Ti_3_C_2_T_x_/SnO_2_ ratios (2.02 wt percent in ETLs) [123].

**Figure 10:**
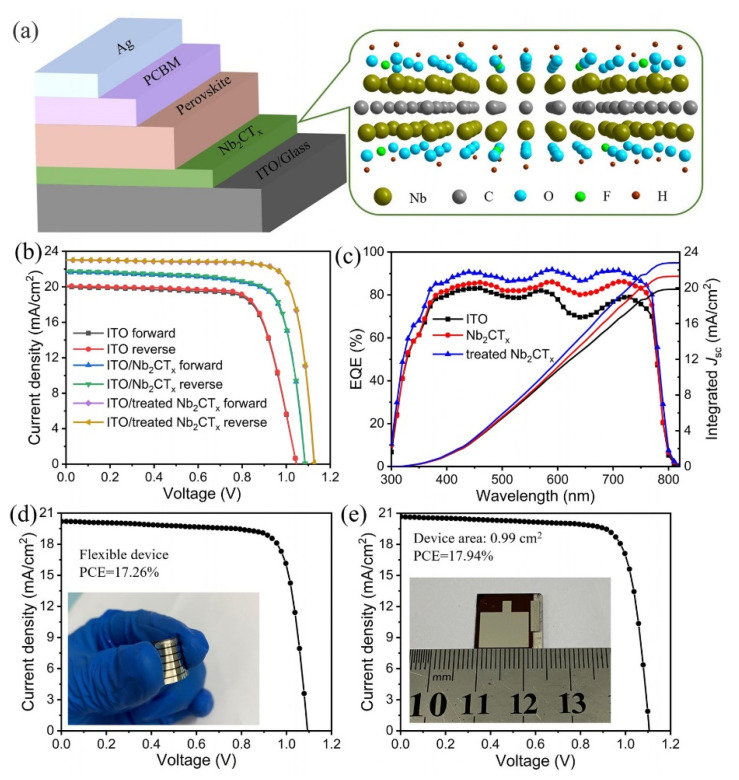
(**a**) The schematic diagram of the device structure and the structure of Nb_2_CT_x_ MXene. (**b**) JV curves of PVSCs measured under different scan directions. (**c**) External quantum efficiency (EQE) and integrated Jsc curves of various PVSCs. JV curves of the flexible (**d**) and large-area (**e**) PVSCs using Nb_2_CT_x_-HTL treated with oxygen plasma. Reprinted with permission from ref. [124]. Copyright 2021 AIP Publishing LLC.

J. Zhang et al. developed the Nb_2_CTx-MXene, which has outstanding photoelectric characteristics and can be utilised as the HTL in fabricating the inverted PVSCs. Enhancing the O-terminated functional groups on the Nb_2_CT_x_ surface, oxygen plasma treatment altered the work function (WF) of Nb_2_CT_x_ HTL. PVSCs with oxygen-plasma-treated Nb_2_CT_x_ HTL have the greatest PCE of 20.74 percent and excellent stability. Figure 10 shows a schematic representation of the device construction as well as the structure of Nb_2_CT_x_ MXene, as seen in Figure 10a. The PVSCs’ current density–voltage curves (JV) are presented in Figure 10b for various scan directions. As demonstrated in Figure 10, the enhanced Jsc is attributed to the greater external quantum efficiency values (EQE) owing to more effective charge separation and collecting efficiency (Figure 10c). The Nb_2_CT_x_-HTL treated with oxygen plasma similarly produces flexible and large-area (0.99 cm^2^) PVSCs with PCE of 17.26 percent and 17.94 percent (Figure 10d,e). Furthermore, employing Nb_2_CT_x_ treated with oxygen plasma as HTL, the flexible and large-area (0.99 cm^2^) PVSCs obtain the greatest PCE of 17.26 percent and 17.94 percent, respectively [124].

Wang et al. used a solution procedure at room temperature to show the potential of Ti_3_C_2_T_x_ Mxene as an ETL for efficient PSCs with traditional design. The authors modified the MXene surface using an oxygen plasma treatment and attempted to establish a link between the surface characteristics and MXene termination groups. The contact angle and topography measurements were used to study the surface tension of MXene and the morphology of the associated perovskite. The PbO interactions between perovskite and MXene were shown by high-resolution XPS spectra, which improved device stability [125].

Yang et al. found a superior match in energy levels between the ETL layer and the perovskite in the case of a hybrid of oxidised and pure Ti_3_C_2_T_x_, with a champion PCE of 18.29 percent, compared to PSCs with pure Ti_3_C_2_T_x_ as ETL, with a PCE of 16.50 percent. The intersection of the baseline with the tangent line of the spectra determined the highest occupied molecular orbital (HOMO) and the highest energy levels, while the results of the UV-Vis absorption spectra calculated the lowest unoccupied molecular orbital (LUMO). The enhanced electron mobility in the ETL, which increases electron transport and decreases hole–electron recombination, is responsible for the improvement in PCE. This research shows that these materials have a lot of promise for use in low-temperature-produced PSC and other solar technologies [126].

To develop a new ZnO/Ti_3_C_2_T_x_ nanohybrid composite film, Hou & Yu utilised Ti_3_C_2_T_x_, a representative of MXene, as an additive in zinc oxide (ZnO). By establishing the Zn–O–Ti bond on the ZnO surface, Ti_3_C_2_T_x_ nanosheets generate new electron transport routes between ZnO nanocrystals and passivates the ZnO surface. As a consequence, the PBDB-T: ITIC based photovoltaic devices with ZnO/Ti_3_C_2_T_x_ ETLs have a power conversion efficiency of 12.20 percent, compared to 10.6 percent for the comparable device utilising pure ZnO as the ETL, which is a 15.53 percent improvement. Furthermore, PM6: Y6-based IPSCs obtain a champion power conversion efficiency of 16.5 percent, compared to 15 percent for the reference device, demonstrating the ZnO/Ti_3_C_2_T_x_—ETL’s applicability [102]. Saranin et al. showed that by utilising MXenes as doping for the forming layers, it is possible to adjust the optoelectronic characteristics of inverted p-i-n-perovskite components. When compared to reference cells, the MXene-based devices had a maximum PCE of over 19% and an average growth of +8%, which is a surprising result, given that the MAPbI_3_-based p-i-n cell used spin-coated NiO [127].

## 4. Conclusions and Prospect

From the discovery of MXene in 2011 up to now, MXene has achieved tremendous technological developments. In 2018, MXene entered into the development of solar cell production by enhancing the effectiveness of energy produced and the stability of solar cells. This review attempts to compile all previously published research on adding MXene into PSCs to enhance operational stability and solar energy collection. According to MXene’s function, the most essential device parameters are given in Tables S1–S3 (Supplementary Materials).

The main conclusions of this work are:

1. Adoption of perovskite solar cells for effective use in solar energy technology due to their good stability against moisture, heat, and light as well as good crystallisation and low density of defects in perovskite films.
2. The use of titanium carbide (Ti_3_C_2_T_x_) in perovskite solar cells resulted in a steady-state energy conversion efficiency of 23.3% and outstanding stability.
3. MXenes combine with other materials to create hybrids and nanocomposites with improved or additional functions. These innovative materials could be used in applications such as renewable energy, energy storage, and conversion.
4. It has become clear to us that the use of a hybrid MXene with carbon nanotubes (m-SWCNTs) can effectively improve the photovoltaic performance of perovskite solar cells due to the presence of hybrid interfacial layers that can reduce defect density and thus improve charge extraction and transfer.
5. From the above tables, it is clear to us that in the last year, the use of MXene as an electron transport layer (ETL) for solar cells has dominated scientific research due to efficient PSCs with conventional design through a solution method at room temperature.
6. All kinds of 2D transition-metal MXenes demonstrated behavior not previously seen in mono-M MXenes, indicating the potential for the use of 2D transition-metal MXenes in a variety of novel applications. Researchers can tune the performance of MXenes for a variety of applications, including nanomagnets, transparent electronics, semiconductors, supercapacitors, and structural materials, by controlling the composition of the 2D transition-metal MXenes phase. This level of control over their composition and structure is unique in the area of 2D materials, and it opens up new avenues for nanomaterial design. The addition of 2D transition-metal MXenes to the category of 2D materials has increased the design options for nanomaterials to satisfy the needs of growing technology.

## Supplementary Materials

The following are available online at https://www.mdpi.com/article/10.3390/nano11102732/s1, Tables S1–S3: Summary of the key parameters for the solar cells employing MXenes.
