# Supplementary material for: A Brief Review of the Role of 2D Mxene Nanosheets toward Solar Cells Efficiency Improvement
Source: Nanomaterials (Basel). 2021 Oct 15;11(10):2732. doi: 10.3390/nano11102732 (PMC8541472; doi:10.3390/nano11102732)
Supplement: Supplementary file 1 [file nanomaterials-11-02732-s001.zip › nanomaterials-1420818-supplementary.pdf]

nanomaterials

Supplementary Materials

A Brief Review of the Role of 2D Mxene Nanosheets toward Solar Cells Efficiency Improvement

Table S1. Summary of the key parameters for the solar cells employing MXenes as hole/electron transport layers Device.

Device architecture	J_{sc} (mA·cm^{-2})	Voc (V)	FF (%)	PCE (%)	Year	Reference
ITO/U-$Ti_3C_2T_x$/PBDB-T:ITIC/Ca/Al (HTL)	15.98	0.89	64	9.02	2019	[1]
ITO/ETL/$CH_3NH_3PbI_3$/spiro-OMeTAD/Ag	23.11	1.07	74	18.29	2021	[2]
ITO/NiO/$MAPbI_3$/PCBM/BCP/Ag	22.88	1.09	77	19	2021	[3]
ITO/SnO_2/perovskite/spiro-MeOTAD/MoO_3/Au	24.96	1.14	0.798	23.3	2021	[4]
FTO/$Ti_3C_2T_x$@TiO2/$C_{s2}AgBiBr_6$/Spiro-OMeTAD/MoO_3/Ag	4.14	0.96	70	2.81	2021	[5]
MXene/m-SWCNTs (2:1)	25.09	1.073	80	21.42	2021	[6]
ITO/UH-$Ti_3C_2T_x$/PBDB-T:ITIC/MoO_3/Al (ETL)	17.36	0.87	60	9.06	2019	[1]

Table S2. Summary of the key parameters for the solar cells employing MXenes as electrodes.

Device architecture	J_{sc} (mA·cm^{-2})	Voc (V)	FF (%)	PCE (%)	Year	Reference
FTO/TiO_2/$MAPbI_3$/$Ti_3C_2T_x$	22.96	0.95	63	13.83	2019	[7]
(Ti_3C_2)/(V_2C)	12.8	0.724	45	7.2	2021	[8]
(ITO/treated Nb_2CT_x/$MAPbI_3$)	23.06	1.128	79.75	20.74	2021	[9]
FTO/c-TiO_2/$CsPbBr_3$/Carbon:CNT:$Ti_3C_2T_x$	7.16	1.357	72.97	7.09	2019	[10]
MXene/CoS composite CE	21.29	0.671	56.6	8.09	2021	[11]
Ti_3C_2 CE-based DSSCs	17.1	0.758	73	8.68	2021	[12]
Ti_3C_2Tx/n+-Si/n-Si/p+-Si/Al_2O_3/SiNx/Ag/Al	36.7	0.54	57.99	11.47	2019	[13]

Table S3. Summary of the key parameters for the solar cells employing MXenes as an additive Device.

Device architecture	J_{sc} (mA·cm^{-2})	Voc (V)	FF (%)	PCE (%)	Year	Reference
FTO/cTiO_2:$Ti_3C_2T_x$/mTiO_2:$Ti_3C_2T_x$/$Ti_3C_2T_x$/$MAPbI_3$:$Ti_3C_2T_x$/Spiro-OMeTAD/Au	23.82	1.09	77.6	20.14	2019	[14]
FTO/SnO_2/$CH_3NH_3PbI_3$/spiro-OMeTAD/Au	23.48	1.12	73.66	19.27	2021	[15]
FTO/SnO_2-TiOxCl_{4-2x}/$CsPbBr_3$:$Ti_3C_2Cl_x$/$Ti_3C_2Cl_x$/carbon	7.87	1.702	82.7	11.08	2021	[16]
Glass/ITO/SnO_2/2D perovskite/Spiro-OMeTAD/Ag	20.87	1.11	67.84	15.71	2021	[17]
ITO/SnO_2/$MAPbI_3$:$Ti_3C_2T_x$/Spiro-OMeTAD/Au	22.26	1.03	76	17.41	2018	[18]
ITO/SnO_2-Ti_3C_2/$MAPbI_3$/Spiro-OMeTAD/Ag	23.14	1.06	75	18.34	2019	[19]
ITO/ZnO:$Ti_3C_2T_x$/PBDB-T:ITIC/MoO_3/Ag	18.63	0.93	70.39	12.2	2020	[20]
ITO/ZnO:$Ti_3C_2T_x$/PM_6:Y_6/MoO_3/Ag	26.38	0.83	75.4	16.51	2020	[20]
ITO/ZnO:$Ti_3C_2T_x$/PTB7:$PC_{71}BM$ /MoO_3/Ag	17.53	0.77	69.33	9.36	2020	[20]

References

1. Yu, Z.; Feng, W.; Lu, W.; Li, B.; Yao, H.; Zeng, K.; Ouyang, J. MXenes with tunable work functions and their application as electron- and hole-transport materials in non-fullerene organic solar cells. *J. Mater. Chem. A* **2019**, *7*, 11160–11169. https://doi.org/10.1039/c9ta01195a.
2. Yang, L.; Kan, D.; Dall'Agnese, C.; Dall'Agnese, Y.; Wang, B.; Jena, A.K.; Wei, Y.; Chen, G.; Wang, X.-F.; Gogotsi, Y.; et al. Performance improvement of MXene-based perovskite solar cells upon property transition from metallic to semiconductive by oxidation of $Ti_3C_2T_x$ in air. *J. Mater. Chem. A* **2021**, *9*, 5016–5025. https://doi.org/10.1039/d0ta11397b.
3. Saranin, D.; Pescetelli, S.; Pazniak, A.; Rossi, D.; Liedl, A.; Yakusheva, A.; Luchnikov, L.; Podgorny, D.; Gostischev, P.; Didenko, S.; et al. Transition metal carbides (MXenes) for efficient NiO-based inverted perovskite solar cells. *Nano Energy* **2021**, *82*, 105771. https://doi.org/10.1016/j.nanoen.2021.105771.
4. Yang, Y.; Lu, H.; Feng, S.; Yang, L.; Dong, H.; Wang, J.; Tian, C.; Li, L.; Lu, H.; Jeong, J.; et al. Modulation of perovskite crystallization processes towards highly efficient and stable perovskite solar cells with MXene quantum dot-modified SnO_2. *Energy Environ. Sci.* **2021**, *14*, 3447–3454. https://doi.org/10.1039/d1ee00056j.
5. Li, Z.; Wang, P.; Ma, C.; Igbari, F.; Kang, Y.; Wang, K.-L.; Song, W.; Dong, C.; Li, Y.; Yao, J.; et al. Single-Layered MXene Nanosheets Doping TiO_2 for Efficient and Stable Double Perovskite Solar Cells. *J. Am. Chem. Soc.* **2021**, *143*, 2593–2600. https://doi.org/10.1021/jacs.0c12739.
6. Bati, A.S.R.; Hao, M.; Macdonald, T.J.; Batmunkh, M.; Yamauchi, Y.; Wang, L.; Shapter, J.G. 1D–2D Synergistic MXene-Nanotubes Hybrids for Efficient Perovskite Solar Cells. *Small* **2021**, *17*, 2101925. https://doi.org/10.1002/smll.202101925.
7. Cao, J.; Meng, F.; Gao, L.; Yang, S.; Yan, Y.; Wang, N.; Liu, A.; Li, Y.; Ma, T. Alternative electrodes for HTMs and noble-metal-free perovskite solar cells: 2D MXenes electrodes. *RSC Adv.* **2019**, *9*, 34152–34157. https://doi.org/10.1039/c9ra06091j.
8. Xu, C.; Zhao, X.; Sun, M.; Ma, J.; Wu, M. Highly effective 2D layered carbides counter electrode for iodide redox couple regeneration in dye-sensitized solar cells. *Electrochim. Acta* **2021**, *392*, 138983. https://doi.org/10.1016/j.electacta.2021.138983.
9. Zhang, J.; Huang, C.; Yu, H. Modulate the work function of Nb_2CT_x MXene as the hole transport layer for perovskite solar cells. *Appl. Phys. Lett.* **2021**, *119*, 033506. https://doi.org/10.1063/5.0057978.
10. Mi, L.; Zhang, Y.; Chen, T.; Xu, E.; Jiang, Y. Carbon electrode engineering for high efficiency all-inorganic perovskite solar cells. *RSC Adv.* **2020**, *10*, 12298–12303. https://doi.org/10.1039/d0ra00288g.
11. Chen, X.; Zhuang, Y.; Shen, Q.; Cao, X.; Yang, W.; Yang, P. In situ synthesis of $Ti_3C_2T_x$ MXene/CoS nanocomposite as high performance counter electrode materials for quantum dot-sensitized solar cells. *Sol. Energy* **2021**, *226*, 236–244. https://doi.org/10.1016/j.solener.2021.08.053.
12. Ahmad, M.S.; Pandey, A.; Rahim, N.A.; Aslfattahi, N.; Mishra, Y.K.; Rashid, B.; Saidur, R. 2-D Mxene flakes as potential replacement for both TCO and Pt layers for Dye-Sensitized Solar cell. *Ceram. Int.* **2021**, *47*, 27942–27947. https://doi.org/10.1016/j.ceramint.2021.06.225.
13. Fu, H.; Ramalingam, V.; Kim, H.; Lin, C.-H.; Fang, X.; Alshareef, H.N.; He, J. MXene-Contacted Silicon Solar Cells with 11.5% Efficiency. *Adv. Energy Mater.* **2019**, *9*, 1–9. https://doi.org/10.1002/aenm.201900180.
14. Agresti, A.; Pazniak, A.; Pescetelli, S.; Di Vito, A.; Rossi, D.; Pecchia, A.; Der Maur, M.A.; Liedl, A.; Larciprete, R.; Kuznetsov, D.V.; et al. Titanium-carbide MXenes for work function and interface engineering in perovskite solar cells. *Nat. Mater.* **2019**, *18*, 1228–1234. https://doi.org/10.1038/s41563-019-0478-1.
15. Zhao, Y.; Zhang, X.; Han, X.; Hou, C.; Wang, H.; Qi, J.; Li, Y.; Zhang, Q. Tuning the reactivity of PbI2 film via monolayer $Ti_3C_2T_x$ MXene for two-step-processed $CH_3NH_3PbI_3$ solar cells. *Chem. Eng. J.* **2021**, *417*, 127912. https://doi.org/10.1016/j.cej.2020.127912.
16. Zhou, Q.; Duan, J.; Du, J.; Guo, Q.; Zhang, Q.; Yang, X.; Duan, Y.; Tang, Q. Tailored Lattice "Tape" to Confine Tensile Interface for 11.08%-Efficiency All-Inorganic $CsPbBr_3$ Perovskite Solar Cell with an Ultrahigh Voltage of 1.702 V. *Adv. Sci.* **2021**, *2101418*, 2101418. https://doi.org/10.1002/advs.202101418.
17. Jin, X.; Yang, L.; Wang, X.-F. Efficient Two-Dimensional Perovskite Solar Cells Realized by Incorporation of $Ti_3C_2T_x$ MXene as Nano-Dopants. *Nano-Micro Lett.* **2021**, *13*, 68. https://doi.org/10.1007/s40820-021-00602-w.
18. Guo, Z.; Gao, L.; Xu, Z.; Teo, S.; Zhang, C.; Kamata, Y.; Hayase, S.; Ma, T. High Electrical Conductivity 2D MXene Serves as Additive of Perovskite for Efficient Solar Cells. *Small* **2018**, *14*, e1802738. https://doi.org/10.1002/smll.201802738.
19. Yang, L.; Dall'Agnese, Y.; Hantanasirisakul, K.; Shuck, C.E.; Maleski, K.; Alhabeb, M.; Chen, G.; Gao, Y.; Sanehira, Y.; Jena, A.K.; et al. SnO_2–Ti_3C_2 MXene electron transport layers for perovskite solar cells. *J. Mater. Chem. A* **2019**, *7*, 5635–5642. https://doi.org/10.1039/c8ta12140k.
20. Hou, C.; Yu, H. ZnO/$Ti_3C_2T_x$ monolayer electron transport layers with enhanced conductivity for highly efficient inverted polymer solar cells. *Chem. Eng. J.* **2021**, *407*, 127192. https://doi.org/10.1016/j.cej.2020.127192.
